# Pharmacist educators in family medicine residency programs: A qualitative analysis

**DOI:** 10.1186/1472-6920-12-74

**Published:** 2012-08-11

**Authors:** Derek Jorgenson, Andries Muller, Anne Marie Whelan

**Affiliations:** 1 College of Pharmacy and Nutrition, University of Saskatchewan, Saskatoon, SK, Canada; 2 Department of Academic Family Medicine, College of Medicine, University of Saskatchewan, Saskatoon, SK, Canada; 3 College of Pharmacy and Department of Family Medicine, Dalhousie University, Halifax, NS, Canada

## Abstract

**Background:**

25-29% of North American family medicine residency programs utilize a pharmacist to teach residents. Little is known about the impact that these pharmacist educators have on residency training. The purpose of this study was to examine the experiences of residents, residency directors and pharmacists within Canadian family medicine residency programs that employ a pharmacist educator to better understand the impact of the role.

**Methods:**

Recruitment from three cohorts (residents, residency directors, pharmacists) within family medicine residency programs across Canada for one-on-one semi-structured interviews followed by thematic analysis of anonymized transcript data.

**Results:**

11 residents, 6 residency directors and 17 pharmacist educators participated in interviews. Data themes were: (1) strong value of the teaching with respect to improved resident knowledge, confidence and patient care delivery; (2) lack of a formal pharmacotherapy curriculum; (3) desire for expansion of pharmacist teaching; (4) impact of teaching on collaboration; (5) impact of teaching on residency program faculty; and (6) lack of criticism of the role.

**Conclusions:**

The pharmacist educator role is valued within residency programs across Canada and the role has a positive impact on several important aspects of family medicine resident training. Suggestions for improvement focused on expanding the teaching role and on implementing a formal curriculum for pharmacist educators to follow.

## Background

Health systems in most developed countries are moving towards a more interprofessional approach to patient care. This new team-based paradigm of care delivery has had a significant impact on the role of pharmacists within primary health systems. Within the last five to ten years, non-dispensing clinical pharmacists have been integrated within many Primary Care Trusts in the United Kingdom [1,2], family medicine teams in North America [3-5] and in similar practice settings around the world [6]. In addition to providing direct patient care services, pharmacists are also commonly involved in the pharmacotherapy education of other health professionals on these teams. This pharmacist educator role is particularly common in family medicine residency programs that utilize a collaborative, interprofessional approach to patient care. Three surveys of family medicine residency programs in the United States found that 25% - 29% of programs reported having a pharmacist involved as a clinical consultant and resident educator [7-9]. In Canada in 1994 only 13.8% of family medicine residency programs reported utilizing the services of a pharmacist educator; this increased to 25.3% in 2011 [10,11]. In all these studies pharmacists were located within the clinic in which the residents trained and had regular informal and formal teaching sessions with the residents for the entire duration of their residency program. Pharmacists used a broad range of instructional methods to teach residents, such as lectures, informal teaching during patient care provision, clinical shadowing and direct mentorship [7-11]. This pharmacist educator role within family medicine residency programs does not appear to be common outside of the United States and Canada, as only one published paper (from Israel) was identified describing a similar service based outside of North America [12].

The limited research that has been published regarding pharmacist educators within family medicine residency programs suggests that the role has a positive impact. Three studies reported either positive resident satisfaction [13] or improvements in resident drug knowledge [14,15] soon after an educational session provided by a pharmacist. An additional qualitative evaluation of an individual pharmacist teaching within a residency program in the United States concluded that the teaching role resulted in changes in resident attitudes towards pharmacists (i.e., residents with better understanding of pharmacist skills) and improved resident drug knowledge [16]. No large multi-centre studies were identified that evaluated the impact of the consistent teaching presence of a pharmacist educator.

Considering the significant resources currently supporting the presence of pharmacist educators in 25-29% of North American family medicine residency programs, and the potential that the role may expand internationally, it is important to determine the extent to which this teaching role impacts family medicine resident training.

The purpose of this study was to examine the perceptions and experiences of residents, residency directors and pharmacist educators regarding the value and importance of pharmacists being involved in teaching family medicine residents about pharmacotherapy.

## Methods

This qualitative study used one-on-one semi-structured interviews with family medicine residents, family medicine residency program directors and pharmacist educators from across Canada and thematic analysis of data to explore the impact of the pharmacist educator role on family medicine resident training. Research ethics approval was obtained from the University of Saskatchewan Research Ethics Board.

### Recruitment and data collection

The websites of the 17 family medicine residency programs in Canada were accessed to identify the contact information of the residency directors. Information not available on the websites was collected by contacting the programs via telephone. Each residency director was emailed and asked which of their core training sites had a pharmacist directly involved in teaching residents. A letter was emailed to residency directors who reported to have a pharmacist educator within their program, asking if the director was willing to take part in a one-on-one telephone interview. Directors were also asked to forward the invitation letter to their pharmacist educator(s) and residents, requesting an interview. Two reminder emails were sent to directors, two and four weeks after the first email, who had not responded.

**Figure 1:**
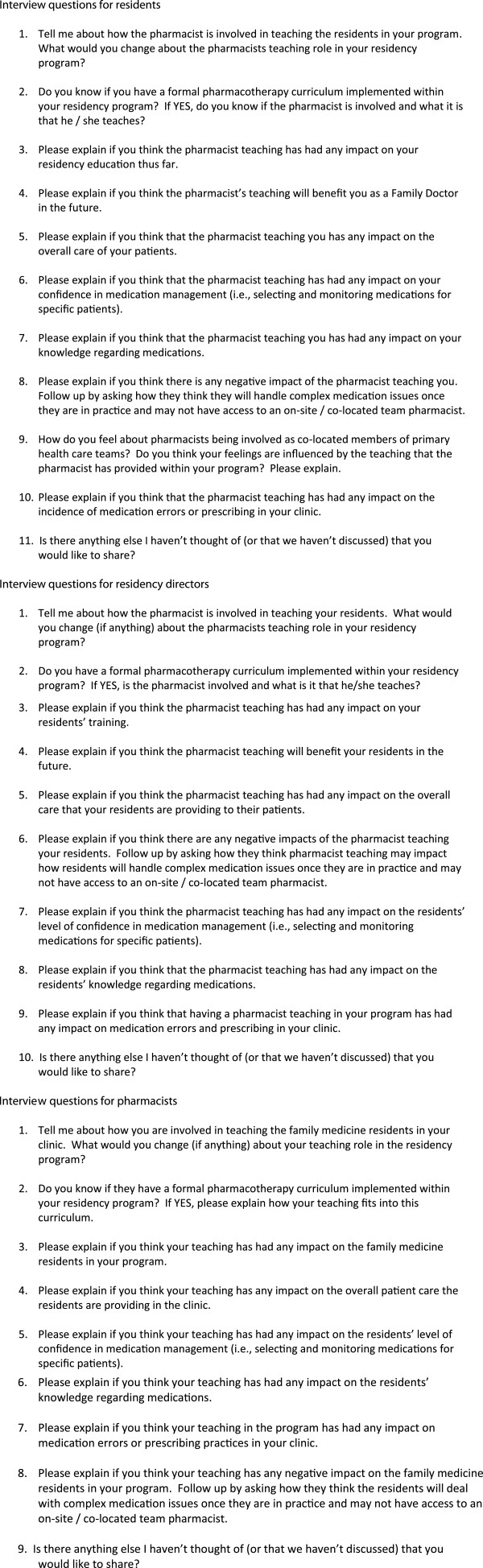
Sample interview guides.

One-on-one telephone interviews were performed and recorded by three different interviewers. A research assistant with previous interviewing experience who was a pharmacy student at the University of Saskatchewan performed the interviews with the residents. A member of the research team (DJ) who is also a pharmacist educator in a family medicine residency program interviewed the pharmacists. A second member of the research team (AM) who is a faculty physician in a family medicine residency program interviewed the directors. This approach of having a learner interview a learner, a pharmacist interview a pharmacist and a physician interview a physician was selected to ensure the interviewers understood the culture within which the interviewees practice, making it easier for them to ask appropriate follow up questions and identify when saturation was reached. The interviewers followed a pre-designed interview guide developed by the research team (Figure 1); however, as these were semi-structured interviews, the interviewers were encouraged to ask additional follow up questions to better clarify individuals’ responses.

We utilized a purposive sampling strategy [17] and endeavored to continue interviewing participants from the three target groups (resident, director, pharmacist) until: (1) there was a reasonable representation of each group from various regions of Canada; and (2) data saturation was observed in all three groups. Data saturation was defined as the point in the data collection when new data no longer brought additional insights to the research question [18,19]. The interviewers met regularly during the interview process to review transcripts and collaboratively determine the saturation point.

### Data analysis

Four members of the research team (one family physician [A.M.]), two pharmacists with experience teaching in family medicine residency programs [D.J., A.M.W.] and a pharmacy student) independently reviewed and analyzed the interview transcripts using both a deductive and inductive approach [20,21]. A standardized template was created for deductive analysis. The template sought to capture data related to potential themes regarding the teaching role that were identified in the literature (resident satisfaction, improved resident drug knowledge, positive attitudes towards pharmacists) and that were hypothesized by the research team (lack of a formal curriculum, improved patient care, confidence in prescribing, reduced medication errors). A section at the end of the template allowed space for a more inductive analysis and further thoughts on the data.

Analysts immersed themselves in the data over multiple readings, making notes in the margins and assigning codes as themes began to emerge and “crystallize” out of the data [18]. The transcripts were analyzed separately for each of the three interview groups (pharmacists, directors, residents). Codes were discussed and debated by the research team members in three separate meetings, using an iterative grounded theory thematic approach to identify common themes [19,21]. Codes identified in these meetings were entered into N-Vivo (version 9.0), a qualitative data analysis and organization software program, to confirm the themes that the research team had manually identified. To confirm the accuracy and completeness of the finalized codes and themes identified by the research team, and as a form of member checking, two family medicine residents who were also interview participants independently reviewed the transcripts and commented on the research teams’ completed and finalized common themes [17-19].

Triangulation was used to confirm and verify the conclusions drawn from the analysis. The aim was to pick triangulation sources (researchers with varied backgrounds, three discreetly different data sources/interviewees, additional analysts external to the research team and data analysis software) that have inherently different biases and strengths, which improves the validity of the conclusions when all of the sources find good convergence [17].

## Results

One hundred residency directors were identified as being responsible for 158 training sites within the 17 family medicine residency programs in Canada (i.e., the 17 programs utilize many different training sites that each employ a director). Eighty-six of the 100 residency directors (86.0%) responded to the email asking which of their training sites had direct involvement of a pharmacist in resident teaching. These 86 directors reported that 25.3% (40 / 158) of the training sites had the direct involvement of a pharmacist in resident teaching. A detailed description of the 40 residency training sites that employ a pharmacist teacher is published elsewhere [11].

Interviews were completed with 11 family medicine residents, six residency directors and 17 pharmacist educators who practiced within the 40 family medicine residency sites that employ a pharmacist educator. Thirty of the 40 training sites were represented by one or more interviewee. Data saturation was observed in all three groups. Although saturation was observed after 12 pharmacist interviews, five additional interviews were in progress when saturation was reached and were therefore included in the analysis. All of the residents were in the second year of their program. There was a reasonable representation in each group from the various regions of Canada (Table 1).

**Table 1:** Regional representation of interviewees

**Region**	**Residents (n = 11)**	**Pharmacists (n = 17)**	**Residency directors (n = 6)**
West^a^	3	4	2
Ontario	5	8	3
Quebec	1	3	1
Atlantic^b^	2	2	0

^a^ British Columbia, Alberta, Saskatchewan, Manitoba.

^b^ New Brunswick, Nova Scotia, Prince Edward Island, Newfoundland and Labrador.

### Themes from deductive analysis

The results of the deductive analysis confirmed the hypotheses that the pharmacist educator role has a positive impact on resident training and that the residency programs do not use a formal pharmacotherapy curriculum to guide pharmacist teaching.

All three interview groups independently confirmed that a formal evaluation of impact of the pharmacist educator role within their residency program had never been performed. Nevertheless, there was a consistently strong message expressed by all three groups that the role had a positive impact on resident training. The residents consistently felt that the teaching role would make them better doctors in the future. The residency directors and residents found this service to be valuable and highly appreciated. Four sub-themes emerged regarding the detailed nature of the positive impact of the pharmacist teaching role: (1) improved patient care; (2) improved drug knowledge; (3) improved confidence; (4) appreciation for the expertise of pharmacists.

1. Improved patient care

All three groups consistently reported that the pharmacist teaching role led to a direct improvement in the quality of the patient care provided by the residents within the clinic.

Resident - “It has changed or modified my approach to the medications I prescribe and to some of the parameters that I am monitoring.”

Resident – “Specific things I have asked the pharmacist about with one patient, I have remembered and that has definitely affected patients that I see later. It is improving care in almost every situation.”

Pharmacist - “…when I am at the hospital, I see the prescriptions from the residents and I can see that they apply the tools or tips that we are giving in the workshops.”

Director – “We think (the teaching) is leading to better care, (but) we never really checked that out. It is just a feeling that we have.”

Director – “Yes I think it definitely leads to better care. (The pharmacist) makes the residents think of the finer details of prescribing and of medications.”

2. Improved drug knowledge

All three interview groups consistently reported that that the residents’ overall drug knowledge improved as a result of the pharmacist educator.

Resident – “I went to a seminar that educated us on different kinds of insulin and…how to administer insulin, which I had no idea about before the seminar.”

Resident – “Our pharmacist has clarified the many over-the-counter medications available and now I can guide patients in terms of what they choose.”

Director – “I am sure it improves resident drug knowledge. A resident had a guy with gout and he remembered what was discussed in rounds the week before, so it sticks with (them).”

3. Improved confidence

All three groups consistently responded that the pharmacist educator improved resident confidence in prescribing and managing complex medication regimens. Residents consistently touched on the impact that this teaching role had on their overall confidence and many provided anecdotes about how the pharmacist directly affected their prescribing practices.

Resident – “If I choose a medication that the pharmacist suggested as their first choice…then I must be doing something right. That is always a confidence builder.”

4. *Appreciation for the expertise of pharmacists*

All three groups observed that, as a result of the pharmacist educator, residents developed an improved understanding of a pharmacists’ expertise and the services that a pharmacist can offer in a primary health care setting. Some residents expressed a sense of regret about losing this pharmacist resource and some had already contemplated how they would ensure they could access similar pharmacist services post-residency.

Pharmacist – “A lot of residents come with preconceived ideas of what a pharmacist does, but here they get regular exposure to me and they start to understand the pharmacists’ skill set and what we can bring to the table in terms of collaborative patient care.”

Resident - “It is always good to know what (pharmacist’s) responsibilities are and what their abilities are so we can use them when we are in practice. I learned that from our pharmacist.”

Resident – “I am going to be sad (when I don’t have access to this pharmacist) and I am not sure what I am going to do, but I have thought about it a few times. I will need to establish a relationship with a community pharmacist I suppose.”

### Themes from inductive analysis

The inductive analysis identified the following themes, which were not anticipated or hypothesized by the research team prior to the study: (1) desire for expansion of pharmacist teaching; (2) impact of the teaching on collaboration; (3) impact of the teaching on faculty; (4) lack of criticism of the role.

1. Desire for expansion of pharmacist teaching

Directors and residents consistently stated that the pharmacist educator role with their residency program should be expanded. No themes emerged regarding additional teaching roles or therapeutic topics that were desired and most appeared to want ‘more of the same’. The residents noted that currently the pharmacists taught using a combination of formal, scheduled lectures and informal, unscheduled discussions while caring for patients. Residents felt that it would be beneficial if the pharmacists teaching role was more ‘formalized’ with more regularly scheduled teaching opportunities.

Director – “We have work for a full time pharmacist, but we don’t have it so we don’t do it. I don’t think there would be anything about the pharmacists’ role I would change. Might increase her load, but not change anything.”

Resident – “I wish I had more interactions with them. I wish we had more formal sessions where we could talk about more difficult patients that come to us.”

Pharmacists did not consistently agree that their teaching role should be expanded. They expressed concern regarding their ability to fit an expanded teaching role into their already busy schedule and the impact this may have on their ability to provide patient care in the clinic.

Pharmacist – “It has been a real balance (trying to teach the residents more). My own professional interest lies more with seeing patients and I think I consider myself more of a clinician than a teacher.”

2. Impact of the teaching on collaboration

All three groups felt that the pharmacist educator had a significant influence on the residents’ willingness and ability to practice in a collaborative, interprofessional setting. Interviewees hypothesized that these improved collaboration skills were a result of the residents spending two years working closely with the pharmacist. This collaborative learning also appeared to have influenced the residents’ future career plans, as many expressed a desire to find a future practice site that used a similar collaborative approach.

Director – “I think most of our residents now would like to practice in a multi-disciplinary situation. Not that there are that many jobs out there for them, but a lot of them now express the desire to have a team approach.”

In addition, residents voiced support for the importance of involving pharmacists on all family medicine teams within the primary health care system and they recognized that this opinion was influenced by their exposure to pharmacist educators in their residency.

3. Impact of the teaching on faculty

Interestingly, residency directors responded that they felt the entire faculty within the family residency program (not only the residents) received similar benefits and value from having the pharmacist educator on the team.

Director – “I also learn from the pharmacist every day. I think she does as much for us (the faculty) as she does for the residents.”

4. Lack of criticism

When the interviewees were asked to describe the positive or negative impact that the pharmacist educator had on resident training, it was notable that not a single negative response was received. Even when interviewees were specifically prompted to think about negative aspects of the teaching role, none offered an example. When asked to comment about the theoretical concern that the presence of a pharmacist educator might lead residents to become reliant on the service and not learn to independently manage complex medications, all three groups responded that this was not a concern. Interviewees felt that since the pharmacists teach in a manner that does not ‘spoon feed’ the residents, it was unlikely that residents would become reliant on this service.

Resident - “I think we would do more harm by retracting the pharmacist component to our training simply because of the theoretical concern that we might use them as a crutch.”

Pharmacist - “I think you have to bear that in mind when you are teaching and when you are interacting with them…to prepare them as much as possible for the environment that they are likely to find themselves in after graduation.”

## Discussion

Despite growing involvement of pharmacist educators in North American family medicine residency programs, the literature is largely devoid of studies evaluating the impact of this role. This first ever pan-Canadian study significantly expands the understanding of the ways in which pharmacist educators affect family medicine resident training and offers suggestions for the future.

No formal evaluations of the pharmacist educator role had been performed in any of the residency programs in which the interviewees practiced. However, it is powerful and noteworthy that the data from all three interview groups consistently identified a range of examples of how this role has a positive impact. The fact that strong convergence was found with respect to the positive impact of this role, after triangulating the data across multiple sources, suggests that these findings are valid and trustworthy. These findings are further strengthened by the fact that our interviewees represented 30 of the 40 family medicine training programs in Canada that employ a pharmacist educator.

All three groups of participants agreed that resident knowledge regarding pharmacotherapy improves when taught by a pharmacist. This is similar to results from previous studies evaluating a single educational session provided by a pharmacist [13-16]. An exciting aspect of this study is the finding that this drug knowledge translates into improvements in resident confidence as well as resident perceptions about the quality of patient care that they provide. This has never been previously documented in the literature and helps to justify the presence of these educators. The success of the pharmacist educator role is likely partially explained by the fact that the pharmacist educator is located within the residency training site. As a result, the pharmacist can tailor teaching topics to the deficiencies and weakness that they personally observe in resident practice and then the residents can immediately apply the information in practice, with convenient access to the pharmacist educator when additional questions arise.

Although no spontaneous criticisms of the pharmacist educator role came forward in this study, interviewees noted that the pharmacists did not use a curriculum to guide their teaching and many residents suggested a more formal pharmacist teaching role with more frequent scheduled teaching interactions. This lack of a formal curriculum is surprising since this teaching role has been documented in the literature as far back as 1981 [8]. In addition, Guidelines for Pharmacotherapy Curricula in Family Medicine Residency Training were published in 1995, recommending that a clinical pharmacist lead the implementation and teaching of a structured and standardized pharmacotherapy curriculum [22]. However, it appears that it has remained very much an informal part of residency curricula in Canada. This seems to be a rather important oversight and residency programs that offer the service should consider the implementation of a formal curriculum for pharmacist educators.

The multiple ways in which this teaching role appears to positively impact residency training is strong evidence for family medicine residency programs that currently employ a pharmacist educator to continue supporting the role. These results also provide an impetus for residency programs without a pharmacist educator, in North America and abroad, to consider incorporating the service. The issue regarding the need to expand the teaching role of pharmacists currently employed as resident educators is much less clear. Residents and directors consistently shared interest in expanding the pharmacist teaching role, yet the pharmacists were hesitant to agree, considering their many other responsibilities within the clinic.

Pharmacist educators were also found to have a positive impact on residents’ ability to work as part of an interprofessional team and influence their career plans to work in a collaborative setting, which is consistent with previous studies in the literature [13-16]. Although this study was limited to examining the role of pharmacist educators, this theme that emerged from the inductive analysis raises the question about the potential role for other health professions to become more involved in teaching medical residents. One can only imagine the impact on a residency program that had several different health professionals involved in teaching.

Overall, the results of this study suggest that the role of the pharmacist educator in family medicine residency training enhances resident application of knowledge into practice and desire to work in collaborative practices. The findings of this study suggest that pharmacists who are currently teaching in family medicine residency programs should continue in their role and that pharmacist educators should be incorporated into more family medicine residency programs. However, the potential implications of these findings on the health human resources challenges of developing and developed countries cannot be ignored. Countries around the world, including Canada, continue to struggle with a high demand and a limited supply of many health professions, including pharmacists. The global health human resource implications of a decision to significantly expand this pharmacist educator role are unknown. It is unclear what the overall health system impact would be if hundreds or thousands of pharmacists left their current practices to teach medical residents, especially if there were not enough pharmacists to move into the vacant positions.

Although this study included programs in Canada, the results should be transferable across North America as residency training programs in Canada and United States share many similarities and participants were recruited from across a nation reflecting a wide range of experiences. It may be more difficult to extrapolate these results to countries that have family medicine residency training programs that significantly differ from those in North America.

Researcher bias is a potentially significant limitation of this study. Each author has significant experience practicing and teaching within family medicine residency programs that utilize a pharmacist educator and would therefore have some degree of bias towards the positive impact of this teaching role. This bias could have influenced the data collection (two of the authors were interviewers) and the analysis (all three authors were involved in data analysis). We attempted to mitigate the impact of this bias by using standardized interview and data analysis templates; transcribing interviews verbatim; involving one external interviewer and three external data analysts; and using data analysis software. Involving researchers with some degree of inherent bias in the collection and analysis of qualitative data can also enhance the quality and completeness of the research, as these individuals come with a precise understanding of the culture and environment in which the research subjects practice.

## Conclusions

This qualitative study found that pharmacist educators in Canadian family medicine residency programs have a positive impact on resident knowledge related to medications, confidence in managing medication and on the overall quality of patient care they provide. Suggestions for improvement focused expanding the pharmacist role and on creating a formal curriculum for the pharmacist to follow.

Future research could focus on measuring the impact of the pharmacist educator role using quantitative measures such as resident exam scores and patient health outcomes. In addition, future studies could attempt to predict the health human resources consequences of expanding the role of pharmacist educators into large numbers of residency programs. Finally, it would be interesting to perform a similar study to this one outside of North America.

## Competing interests

The authors of this paper have no competing interests to declare.

## Authors contributions

DJ made substantial contributions to the conception and design of this study and was substantially involved in the acquisition, analysis and interpretation of data. DJ took the lead in drafting and revising the manuscript and gave final approval of the version to be published. AM made substantial contributions to the conception and design of this study and was involved in the acquisition, analysis and interpretation of data. AM assisting in revising the manuscript and gave final approval of the version to be published. AMW made substantial contributions to the conception and design of this study and was involved in the acquisition, analysis and interpretation of data. AMW assisting in revising the manuscript and gave final approval of the version to be published. All authors read and approved the final manuscript.

## Pre-publication history

The pre-publication history for this paper can be accessed here:

http://www.biomedcentral.com/1472-6920/12/74/prepub
